# Deviant cortical sulcation related to schizophrenia and cognitive deficits in the second trimester

**DOI:** 10.1515/tnsci-2020-0111

**Published:** 2020-07-15

**Authors:** Michael Lloyd MacKinley, Priyadharshini Sabesan, Lena Palaniyappan

**Affiliations:** Robarts Research Institute & The Brain and Mind Institute, University of Western Ontario, Room-A2/636, Prevention and Early Intervention Program for Psychoses, Victoria Hospital, 800, Commissioners Road, London, Ontario, Canada; Schulich School of Medicine and Dentistry, Department of Neuroscience, University of Western Ontario, London, Ontario, Canada; Department of Psychiatry, University of Western Ontario, London, Ontario, Canada; Lawson Health Research Institute, London, Ontario, Canada

**Keywords:** sulcation, gyrification, schizophrenia, neurodevelopment, neurocognition

## Abstract

**Objectives:**

Aberrant cortical development, inferred from cortical folding, is linked to the risk of schizophrenia. Cortical folds develop in a time-locked fashion during fetal growth. We leveraged this temporal specificity of sulcation to investigate the timing of the prenatal insult linked to schizophrenia and the cognitive impairment seen in this illness.

**Methods:**

Anatomical MRI scans from 68 patients with schizophrenia and 72 controls were used to evaluate the sulcal depth of five major invariable primary sulci representing lobar development (calcarine sulcus, superior temporal sulcus, superior frontal sulcus, intraparietal sulcus and inferior frontal sulcus) with formation representing the distinct developmental periods.

**Results:**

A repeated-measure ANOVA with five sulci and two hemispheres as the within-subject factors and gender, age and intracranial volume as covariates revealed a significant effect of diagnosis (*F*[1,134] = 14.8, *p* = 0.0002). Control subjects had deeper bilateral superior temporal, right inferior frontal and left calcarine sulci. A deeper superior frontal sulcus predicted better cognitive scores among patients.

**Conclusion:**

Our results suggest that the gestational disruption underlying schizophrenia is likely to predate, if not coincide with the appearance of calcarine sulcus (early second trimester). Nevertheless, the burden of cognitive deficits may relate specifically to the aberrant superior frontal development apparent in late second trimester.

## 1 Introduction

An emerging body of evidence implicates aberrations in fetal cortical development to cognitive and mental health outcomes later in life [1,2]. To date, individuals for whom *in utero* developmental changes were recorded have not been followed up until the emergence of psychosis. Very large prospective cohorts are required for this purpose, given the later emergence and low incident rates of schizophrenia.

The primary sulci of the human brain follow a course of programmed progressive development that occurs in a time-locked fashion and is highly sensitive to fetal disruptions [1]. The development of the primary sulci defining lobar development (calcarine sulcus, superior temporal sulcus, superior frontal sulcus, intraparietal sulcus and inferior frontal sulcus) is so precise in the developing fetuses (with emergence at 16, 23, 25, 26 and 28 weeks, respectively [1]) that these structures can be used to estimate gestational age and brain maturation [2]. Studying the location of aberrations in cortical folding in adult life can serve as a window to the time-locked disruptions suffered by the developing cortical architecture [3].

Preterm birth, known to be linked to aberrant *in utero* cortical development, is associated with defects in cortical folding that appear to persist in adult life [4]. Aberrant cortical folding in adults related to preterm birth relates to negative cognitive and mental health outcomes [4]. Clinically, atypical development in the primary sulci has been associated with sensory and information processing deficits among patients with schizophrenia [5,6,7,8,9], potentially contributing to the development of both cognitive deficits and psychotic symptoms. While there is a growing literature of cortical sulcal and gyral morphology in schizophrenia [10,11], the likely timing of embryonic/fetal disruption in schizophrenia continues to be unknown, with prenatal immune models implicating both second and third trimester insults as risk factors [12], and maternal stress and malnourishment models implicate third trimester insults [12,13]. Prenatal insults not only associate with the risk of schizophrenia but also influence the severity of the eventual illness (e.g., the presence of cognitive deficits [14]).

The relationship between cognitive deficits and the clinical expression of schizophrenia has been well studied over the years. Extant results suggest a notable independence in the course of the cognitive and non-cognitive symptom expression [15]. In particular, cognitive symptoms arise much earlier than psychotic symptoms, do not respond to treatment, and continue despite the improvement in psychosis [16]. Furthermore, considerable variability exists in the degree of cognitive deficits, with not all patients exhibiting cognitive features that relate to poor prognosis [16]. Finally, there is no diagnosis-specific pattern in the nature and the course of cognitive deficits in schizophrenia [17]. The genetic correlation between cognitive impairment and schizophrenia is also observed to be low [18]. This raises the important question of whether the embryonic/fetal disruption that relate to the cognitive deficits of schizophrenia occurs at a specific time point, independent of the disruption that relates to the illness itself. If this is the case, then individuals with schizophrenia who have a short period of time-locked developmental disruption of the cortex may have less cognitive deficits; while those with a protracted developmental defect *in utero* will be more cognitively impaired. We hypothesized that patients with schizophrenia, when compared to healthy controls, will show disrupted morphology across various sulci that develop throughout the second and early third trimesters, but patients with severe cognitive deficits will show more pronounced defects affecting the later developing sulci. To this end, we compared sulcal depth of five major primary sulci – calcarine, superior temporal, superior frontal, intraparietal, and inferior frontal – that are anatomically district, with temporal specificity in their gestational appearance based on Chi and colleagues [1], among healthy controls and schizophrenia patients and assessed their relationship to cognitive performance in patients.

### 1.1 Patients

In all, 68 patients with schizophrenia or schizoaffective disorder and 72 healthy controls from the National Institute of Health Centre for Biomedical Research Excellence were included in the analysis. Patients were excluded based on the history of neurological disorder, intellectual disability, severe head trauma, or substance abuse or dependence within the last 12 months.


**Ethical approval:** The research related to human use has been complied with all the relevant national regulations, institutional policies, and in accordance with the tenets of the Helsinki Declaration and has been approved by the authors’ institutional review board or equivalent committee.
**Informed consent:** Informed consent has been obtained from all individuals included in this study.

### 1.2 Method

Diagnostic information was collected using the Structured Clinical Interview used for disorders in the diagnostic and statistical manual of mental disorders [19]. Seven domains of cognition (speed of processing, attention/vigilance, working memory, verbal learning, visual learning, reasoning/problem-solving and social cognition) were assessed using the Measurement and Treatment Research to Improve Cognition in Schizophrenia (MATRICS) battery [18].

The MRI data were collected on a Siemens 3T TIM Trio scanner. A multi-echo Magnetization Prepared RApid Gradient Echo (MPRAGE) (Multi-Echo) sequence was used [20]. Mean sulcal depth was measured using BrainVISA from the sulci identified using the morphologist interface of BrainVISA 4.5 with default settings. Following the construction of three-dimensional models of cortical folds, various sulci were automatically classified using a probabilistic algorithm with maximum depth computed for each identified sulcus. The identified sulci were visually inspected by two raters to ensure that the boundaries are in accordance with Ono’s Atlas of Cerebral Sulci [21].

### 1.3 Statistical approach

The group differences for age, gender, handedness, intracranial volume and MATRICS scores (composite and domain scores) were assessed using SPSS via independent samples *t* tests or chi-square tests where appropriate. Repeated-measures ANOVA were used with the five sulci of interest and two hemispheres as the within-subject factors and gender, age and intracranial volume as covariates to assess for the differences between healthy controls and patients. Parameter estimates were sought to assess the direction of group differences and covariate effects. Five non-collinear factors representing the five bilateral sulci were obtained using varimax rotation with Kaiser normalization using the principal component analysis and using multiple regression were related to the overall MATRICS standardized composite score in patients.

## 2 Results

No statistically significant differences were identified on gender, age, handedness or intracranial volume between patients and controls, but as expected patients had lower scores on the MATRICS composite score as well as several specific cognitive domains including processing speed, attention vigilance, working memory and problem-solving (Table 1).

**Table 1:** Group differences in cognitive and demographic features

Variable	Healthy control *n* = 72	Patients *n* = 68
Gender (M/F)	51/21	55/13
Age, M (SD)	35.87 (11.74)	38.49 (13.86)
Handedness (right/left/both)	69/1/2	57/9/2
Intracranial volume in milliliters, M (SD)	1511.26 (141.58)	1543.51 (157.97)
MATRICS composite score	49.97 (8.31)	32.02 (13.42)*
Processing speed, M (SD)	54.12 (8.94)	35.48 (12.21)*
Attention vigilance, M (SD)	48.57 (9.23)	37.56 (13.37)*
Working memory, M (SD)	50.86 (10.22)	40.84 (12.84)*
Verbal learning, M (SD)	45.88 (8.98)	38.91 (8.89)
Visual learning, M (SD)	45.25 (10.16)	35.96 (11.97)
Problem-solving, M (SD)	55.37 (8.44)	44.50 (11.55)*
Social cognition, M (SD)	51.46 (10.04)	41.91 (10.46)

^*^Indicates significant difference based on independent *t* test at *p* < 0.05.

The ANOVA showed a significant between-subject effect for diagnosis (*F*[1,134] = 14.8, *p* = 0.0002), with significant effects also identified for gender (*F*[1,134] = 7.4, *p* = 0.007, sulcal depth for females > males) and age (*F*[1,134] = 4.5, *p* = 0.035; reduced depth with increasing age). Parameter estimates revealed a significant effect of diagnosis (controls > patients) for left superior temporal (*t* = 3.2, *p* = 0.002), right superior temporal (*t* = 2.8, *p* = 0.006), right inferior frontal (*t* = 2.7, *p* = 0.007) and left calcarine (*t* = 2.2, *p* = 0.03) sulci.

Five orthogonal factors emerged from the 10 sulcal depth metrics, with each factor having a loading of >0.8 for the bilateral sulci that they represented, with no similar loading from any other sulci. Thus, to study the relationship with cognition, we extracted five non-collinear factors, each representing the bilateral sulcal depth of a single sulcus. As age and gender had significant effects on the model, we used them as covariates for the multiple regression analysis relating MATRICS total score to the five orthogonal sulcal factor scores. The depth of the superior frontal sulcus was the only predictor of the variation in the cognitive score (*t* = 2.88, *p* = 0.006; all other predictors *p* > 0.17; overall model *F*[7,52] = 2.15, *p* = 0.055). Patients with a deeper superior frontal sulcus had higher composite cognitive scores. A similar predictive model for MATRICS composite scores was not significant in healthy controls (overall model *F*[7,49] = 1.56, *p* = 0.17).

## 3 Discussion

**Figure 1:**
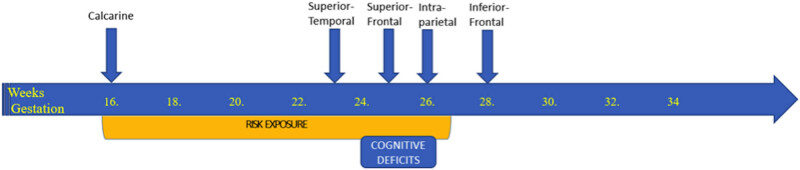
Timeline of the emergence of the five primary sulci under study and the posited risk exposure.

These findings suggest that developmental disruptions predominantly affect the morphology of the frontal, temporal and occipital lobes’ cortical disruption in patients with schizophrenia, with the onset likely to predate or coincide with the appearance of calcarine sulcus (i.e., 16 weeks, early second trimester). In contrast to the illness-related developmental deviation, the burden of cognitive deficits seen among patients may relate specifically to aberrant superior frontal development occurring in late second trimester (Figure 1).

While sulcal depth is fairly constant in early adult life, age-related changes have been reported in adolescence (0.3%/year; age 11–17) [22] and later life, especially affecting the superior frontal sulcus [23]. These changes are considerably smaller in magnitude (<10 times) when compared to age-related changes in sulcal width [24] and other morphometric features. We had age-matched case–control groups and adjusted for age in our regression analysis. Our observations relating schizophrenia to earlier aberrations in development (∼16 weeks), while the cognitive deficits of schizophrenia to later development (∼25 weeks) raises an interesting question. The transient but time-locked insults to the developing brain may be pathogenic; while sustained or repeated insults (that extend to later part of development) may lead to a more severe form of illness characterized by cognitive deficits. Given the low genetic correlation between cognitive impairment and schizophrenia, our results support the notion that the development of these two features may be somewhat independent [18], at least on the timescale of cortical development.

While most of the sulcal morphology shows symmetric heritability, superior temporal sulcus is known to have an asymmetric pattern of heritability in healthy subjects [25,26]. This asymmetry in heritability has led to the inference that language-related genetic control might play a distinct role in the left superior temporal sulcal morphology [26]. Thus, a pathological perturbation restricted to left superior temporal cortex morphology may indicate language-related genetic abnormalities, while bilateral morphological disruption may indicate either a loss of specificity of language-related genetic control of the superior temporal region or a non-genetic pathological mechanism operating bilaterally. In this context, the bilateral reduction in superior temporal sulcal depth observed in our study raises the possibility of an environmental origin of symmetric nature for the apparent *in utero* malformation seen in schizophrenia. Relevant animal models of *in utero* risk exposure are required to address this question.

In summary, our results support the view that deviant cortical sulcation related to schizophrenia likely originates earlier and prevails through the second trimester, with frontal sulcal morphology playing a key role in cognitive deficits seen in this illness. These results need larger scale replication, extension to drug-naive samples and experimental studies in animal models for further validation.
